# Deep Learning Methods for Remote Heart Rate Measurement: A Review and Future Research Agenda

**DOI:** 10.3390/s21186296

**Published:** 2021-09-20

**Authors:** Chun-Hong Cheng, Kwan-Long Wong, Jing-Wei Chin, Tsz-Tai Chan, Richard H. Y. So

**Affiliations:** 1 Department of Computer Science, The Hong Kong University of Science and Technology, Clear Water Bay, Kowloon, Hong Kong, China; chchengak@connect.ust.hk; 2 PanopticAI, Hong Kong Science and Technology Parks, New Territories, Hong Kong, China; jwchin@connect.ust.hk (J.-W.C.); ttchanac@connect.ust.hk (T.-T.C.); rhyso@ust.hk (R.H.Y.S.); 3 Department of Bioengineering, The Hong Kong University of Science and Technology, Clear Water Bay, Kowloon, Hong Kong, China; 4 Department of Industrial Engineering and Decision Analytics, The Hong Kong University of Science and Technology, Clear Water Bay, Kowloon, Hong Kong, China

**Keywords:** noncontact monitoring, heart rate measurement, remote photoplethysmography, rPPG, deep learning

## Abstract

Heart rate (HR) is one of the essential vital signs used to indicate the physiological health of the human body. While traditional HR monitors usually require contact with skin, remote photoplethysmography (rPPG) enables contactless HR monitoring by capturing subtle light changes of skin through a video camera. Given the vast potential of this technology in the future of digital healthcare, remote monitoring of physiological signals has gained significant traction in the research community. In recent years, the success of deep learning (DL) methods for image and video analysis has inspired researchers to apply such techniques to various parts of the remote physiological signal extraction pipeline. In this paper, we discuss several recent advances of DL-based methods specifically for remote HR measurement, categorizing them based on model architecture and application. We further detail relevant real-world applications of remote physiological monitoring and summarize various common resources used to accelerate related research progress. Lastly, we analyze the implications of research findings and discuss research gaps to guide future explorations.

## 1. Introduction

Human vital signs, such as heart rate (HR), body temperature (BT), respiratory rate (RR), blood oxygen saturation (SpO2), heart rate variability (HRV), and blood pressure (BP), are common indicators used for monitoring the physiological status of the human body [1,2,3,4]. They can be used to estimate and analyze a person’s physical health, detect possible diseases, and monitor recovery. In particular, closely monitoring a person’s HR can enable early detection and prevention of cardiovascular problems, such as atherosclerosis (heart block) and arrhythmia (irregular heart rate) [5].

Photoplethysmography (PPG) is a common method for measuring HR. It utilizes a light source and photodetector to measure the volumetric changes of blood vessels under the skin [6,7]. As the light source illuminates the tissue, small variations in reflected or transmitted light intensity from blood flow are captured by the photodetector, yielding the so-called PPG signal [7]. The absorption of light follows the Beer–Lambert law, which states that the light absorbed by blood is proportional to the penetration of light into the skin and the concentration of hemoglobin in the blood [8]. During the cardiac cycle, minute variations in hemoglobin concentration cause fluctuations in the amount of light absorbed by the blood vessels, resulting in changes of skin intensity values. Pulse oximeters are commonly used for non-invasive measurement of these slight variations in the skin through PPG. However, as with other wearables and contact-based devices (e.g., smartwatches), they are unsuitable for monitoring newborns or patients with fragile skin [9,10]. Furthermore, long-term monitoring may lead to discomfort and even the risk of skin infections [11]. As a result, remote PPG (rPPG) methods have emerged as an attractive alternative.

**Figure 1:**
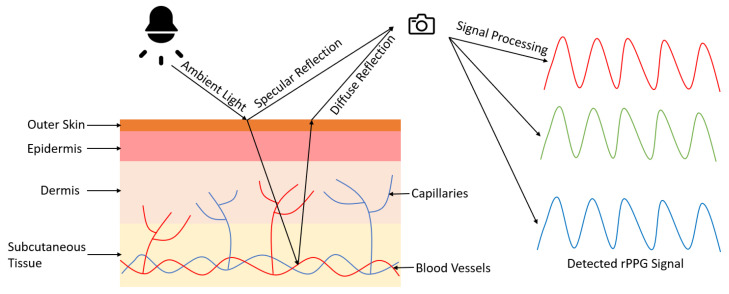
Principle of remote photoplethysmography (rPPG) based on the dichromatic reflection model (DRM). The digital camera captures the specular and diffuse reflection from ambient light. The specular reflection contains surface information that does not relate to physiological signals, while the diffuse reflection is modulated by blood flow. The rPPG signal can be obtained from further signal processing.

During the last decade, rPPG methods have gained significant traction. In rPPG, a digital camera (e.g., webcam, standard RGB camera, near-infrared camera) functions as a photodetector that captures subtle color changes of the skin; ambient light typically serves as the light source [12]. Figure 1 illustrates the principle of rPPG with the dichromatic reflection model (DRM) [13]. According to the DRM, the signals captured by the digital camera are a combination of specular reflections (surface reflections) and diffuse reflections (body reflections). Specular reflections occur at the interface of the incident light and the skin, which do not contain meaningful physiological signals. Thus, rPPG methods utilize signal processing techniques to separate the specular reflections and extract the diffuse reflections associated with the underlying signals of interest. The ability for contactless measurement can significantly reduce monitoring costs and enable applications where traditional contact sensors would be suboptimal [14]. However, while rPPG technology will undoubtedly play a pivotal role in the future of digital healthcare, the extracted signals are inherently much weaker and require meticulous processing.

Verkruysse et al. [15] was the initial research that used a consumer-level camera with ambient light for measurement of rPPG signals. In their work, the green channel was found to contain the most significant PPG signal. Poh et al. [16] applied a blind source separation (BSS) technique, independent component analysis (ICA), on the recorded RGB color channels from a webcam to recover HR. Lewandoska et al. [17] applied a similar method, principal component analysis (PCA), which reduced the computational complexity while achieving a similar accuracy to ICA. However, these methods are subject to motion artifacts. To improve the motion robustness of the rPPG model, a chrominance-based approach (CHROM) was proposed [18]. In this approach, the dichromatic reflection model was used to describe the light reflected from the skin as specular and diffuse reflection components [19]. De Haan and van Leest [20] defined a blood-volume pulse vector, which represents the signature of blood volume change, to identify the subtle color changes due to the pulse from motion artifacts based on RGB measurement. Later, Wang et al. [21] proposed a data-driven algorithm, spatial subspace rotation (2SR), to estimate a spatial subspace of skin pixels and evaluate its temporal rotation to measure HR. Wang et al. [13] further proposed a plane-orthogonal-to-skin (POS) algorithm that defines a projection plane orthogonal to skin tone in the RGB space to extract the pulse signal. Further information about early conventional rPPG methods can be found in the following surveys [14,22,23].

**Figure 2:**
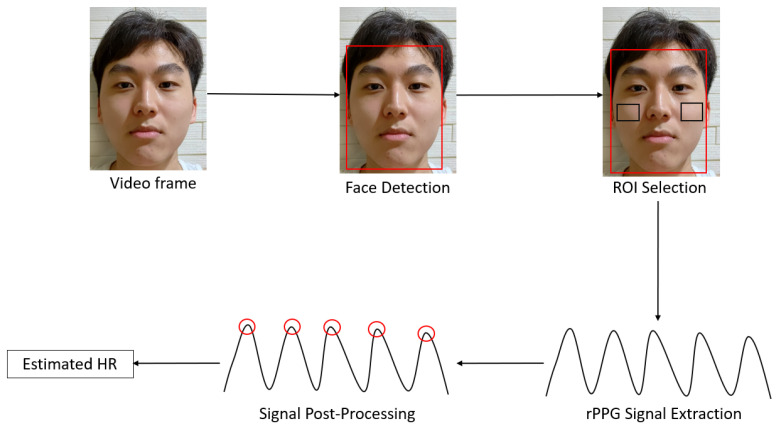
General framework of conventional methods for remote heart rate (HR) measurement. Face detection (e.g., Viola and Jones algorithm) is performed on the video frames, resulting in the red bounding box on the face. Next, regions of interest (ROIs) such as the cheeks marked by the black boxes are selected within the face box. The rPPG signal is extracted from the pixels within the ROIs. Lastly, post-processing techniques, such as frequency analysis (e.g., Fourier transform) and peak detection, are applied on the extracted signal to estimate HR.

Most conventional methods for remote HR measurement follow a similar framework as shown in Figure 2. Firstly, a digital camera captures a video recording of the subject. Next, a face detection algorithm, such as the Viola and Jones algorithm [24], is applied to obtain the bounding box coordinates of the subject’s face. This is followed by selecting regions of interest (ROIs), such as the cheeks, to obtain an area that contains a strong signal. The pixels within the ROI(s) are used for rPPG signal extraction and HR is estimated by further post-processing, which typically involves frequency analysis and peak detection.

As with many computer vision and signal processing applications, DL methods have shown promise in mapping the complex physiological processes for remote HR measurement. While many review papers have discussed the conventional techniques for non-contact physiological monitoring [10,12,14,23], there is limited emphasis on DL methods, despite their popularity in the research community. The number of research papers utilizing DL methods for remote HR measurement has increased year after year and is expected to grow continuously. Our paper aims to provide researchers with an extensive review of DL approaches for remote HR measurement and an improved understanding of their benefits and drawbacks.

In the following sections of this paper, we categorize DL approaches for remote HR measurement as end-to-end and hybrid DL methods. We proceed to classify them based on model architecture and critically analyze their methods. We then discuss the real-world applications that benefit from this technology and introduce some common resources, including toolboxes, datasets, and open challenges for researchers in this field. Finally, we analyze the current knowledge gaps and suggest future directions for research.

## 2. End-to-End Deep Learning Methods

**Figure 3:**
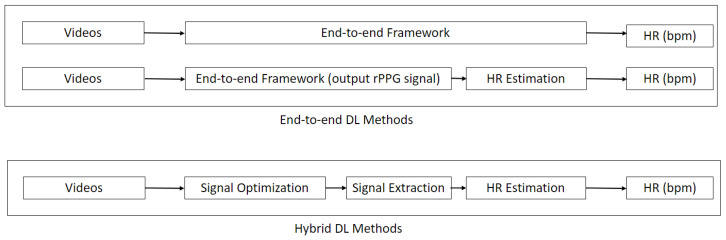
Schematic diagram of end-to-end deep learning (DL) methods and hybrid DL methods. End-to-end DL methods directly output the HR or rPPG signal with a single model, while hybrid DL methods utilize DL techniques at various stages.

In this section, we detail the end-to-end DL approaches for remote HR measurement. We classify a method as end-to-end if it takes in a series of video frames as input and directly outputs the HR without any intermediate steps. Since many DL methods are designed to output the rPPG signal, these are also grouped in the same category for subsequent analysis (Figure 3). As shown in Table 1, the methods are further classified based on the type of DL technique used. While end-to-end DL methods are indisputably great tools due to their straightforward model optimization process, they require enormous amounts of training data and are difficult to validate. More work needs to be done on the interpretation of such models for translation to clinical application [25].

### 2.1. 2D Convolutional Neural Network (2D CNN)

Špetlík et al. [26] proposed an end-to-end HR estimation approach, where the output of the model was a single scalar value of the predicted HR. HR-CNN is a two-step CNN that contains an extractor and an HR estimator. The 2D CNN extractor was trained to maximize the signal-to-noise ratio (SNR) in order to extract the rPPG signal from a sequence of video frames. Then, the extracted rPPG signal was fed into the HR estimator to output the predicted HR value, where the training process minimized the mean absolute error (MAE) between the predicted and ground truth HR. Špetlík et al. [26] claimed that their proposed method better addressed video compression artifacts, where most conventional rPPG signal extraction methods fail. They validated it on three public datasets, as well as proposed a new challenging dataset (ECG-Fitness) which contained different motions and lighting conditions.

**Figure 4:**
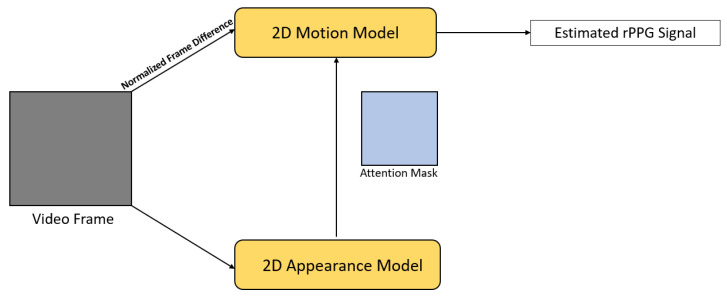
Architecture of DeepPhys [27].

DeepPhys [27] is a VGG-style 2D CNN that jointly trained a motion and appearance model (Figure 4). The motion model took the normalized difference between adjacent frames as an input motion representation; it is built on top of the dichromatic reflection model for modeling motions and color changes. The appearance model guided the motion model to learn motion representation through an attention mechanism. The network learned soft-attention masks from the original video frames and allocated higher weights to skin areas with stronger signals. This attention mechanism also enabled the visualization of the spatio-temporal distribution of physiological signals. With the motion representation and attention mechanism, Chen and McDuff [27] claimed that physiological signals under different lighting conditions can be better captured, being more robust to illumination changes and subject motion.

MTTS-CAN [28] is an improvement built on top of DeepPhys [27]. MTTS-CAN captured temporal information through the introduction of a temporal shift module (TSM) [36]. TSM allowed information exchange among neighboring frames and avoided expensive 3D convolution operations by shifting chunks in the tensor along the temporal axis. In addition, the input of the appearance model was a frame obtained by performing averaging adjacent multiple frames rather than the original video frame. Furthermore, it estimated HR and RR simultaneously by using a multi-task variant. Since this network was completely based on 2D CNNs, it only took 6 ms per frame for on-device inference, which demonstrated its potential of being utilized in real time applications.

### 2.2. Spatio-Temporal Network—3D Convolutional Neural Network (3D CNN)

As 2D CNNs only take spatial information of video frames into account, researchers have proposed different 3D CNN frameworks to also make use of the temporal information contained in the videos. These so-called spatio-temporal networks (STNs) can provide a more comprehensive representation of the spatial and temporal information of the physiological signals in the video stream.

**Figure 5:**
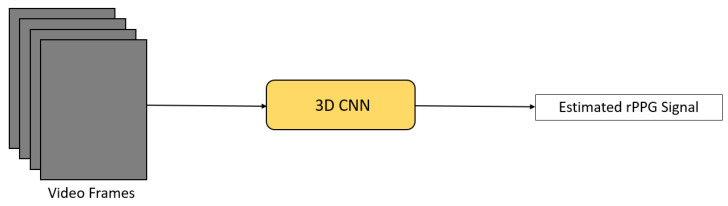
Architecture of 3D CNN PhysNet [29].

Three-dimensional CNN PhysNet [29] is an end-to-end STN aimed at locating the peak of every individual heartbeat (Figure 5). It is able to estimate both HR and HRV accurately, allowing more complicated applications, such as emotion recognition. It took the original RGB video frames as input and directly output the final rPPG signal. In addition, it utilized the negative Pearson correlation as the loss function in order to have higher trend similarity and fewer peak location errors.

**Figure 6:**
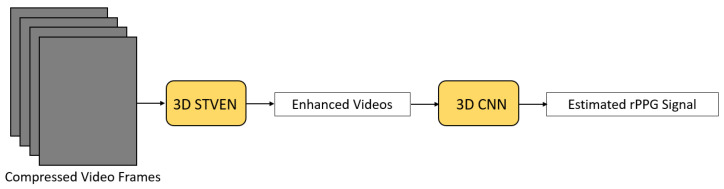
Architecture used in Yu et al. [30].

Yu et al. [30] proposed a two-stage end-to-end STN to not only estimate the rPPG signal but also to overcome the problem of highly compressed facial videos (Figure 6). Compressed facial videos were fed into a spatio-temporal video enhancement network (STVEN) to improve the quality of the videos while retaining as much information as possible. The enhanced videos were further fed into a spatio-temporal 3D CNN (rPPGNet) to extract the rPPG signal. Inside rPPGNet, an attention mechanism was applied to obtain dominant rPPG features from skin regions. rPPGNet is able to operate individually for rPPG signal extraction but can be trained jointly with STVEN to achieve better performance. Yu et al. [30] claimed that rPPGNet is able to recover better rPPG signals with curves and peak locations for accurate HR and HRV estimation.

**Figure 7:**
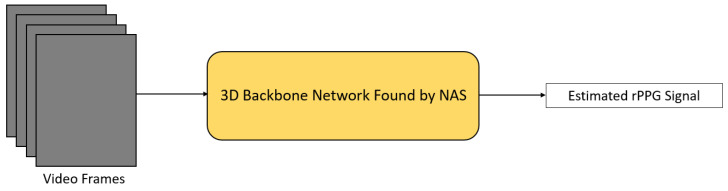
Architecture of AutoHR [31].

Yu et al. [31] utilized neural architecture search (NAS) to automatically find the best-suited backbone 3D CNN for rPPG signal extraction (Figure 7). In their research, a special 3D convolution operation, namely temporal difference convolution (TDC), was designed to help track the ROI and improve the robustness in the presence of motion and poor illumination. Then, NAS was performed based on two gradient-based NAS methods [37,38] in order to form a backbone network for rPPG signal extraction. Two data augmentation methods were proposed, as well, in order to prevent data scarcity.

Hu et al. [32] designed a novel facial feature extraction method in order to avoid extracting redundant information from video segments and to enhance long-range video temporal modeling. A 3D CNN was used to extract facial features of the input video frames. Next, aggregation functions were applied to incorporate long-range spatio-temporal feature maps into short segment spatio-temporal feature maps. These feature maps were then fed into a signal extractor with several spatio-temporal convolution [39] to extract the rPPG signal. A spatio-temporal strip pooling method and an attention mechanism were further applied to the extracted rPPG signal to accommodate head movement and avoid ignoring important local information.

Zhang et al. [33] proposed an efficient multi-hierarchical convolutional network to perform estimation quickly, where only 15 s of face video was required for effectively reconstructing the rPPG signal and estimating HR. A three-layer 3D CNN was used to extract low-level facial feature maps from RGB face videos. These feature maps were passed to a spatio-temporal stack convolution module for deeper feature extraction and generation of a high-level feature map. Channel-wise feature extraction was then performed on the high-level feature map to produce a channel-wise feature map. A skin map was also generated based on low-level feature maps for emphasizing skin regions with stronger signals. Next, a weight mask was constructed by performing feature fusion on the skin map and the channel-wise feature map. Finally, the high-level feature map was multiplied by the weight mask by channels and was fed into a rPPG signal extractor.

**Figure 8:**
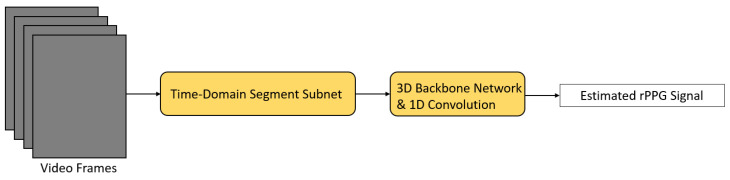
Architecture of ETA-rPPGNet [34].

ETA-rPPGNet [34] is another network aimed at dealing with the problem of extracting redundant video information (Figure 8). In this network, a time-domain segment subnet was designed to model the long-range temporal structure of the video. Split video segments were passed to different subspace networks of this subnet to extract facial features. Then, an attention mechanism was applied to learn important spatial features. Next, an aggregation function was used to aggregate the temporal context in order to cut down redundant video information and a feature map was obtained in each individual subspace network. These individual feature maps were concatenated and fed into the backbone network for rPPG signal extraction. Inside the backbone network, an attention module was also added for eliminating different noise (e.g., head movement, illumination variation). Finally, the extracted rPPG signal was further processed by a 1D convolution operation to model the correlation held in the local time domain effectively.

### 2.3. Spatio-Temporal Network—2D Convolutional Neural Network + Recurrent Neural Network (2D CNN + RNN)

Researchers have also designed another type of spatio-temporal network, which is the combination of 2D CNN for spatial information and RNN for temporal context.

**Figure 9:**
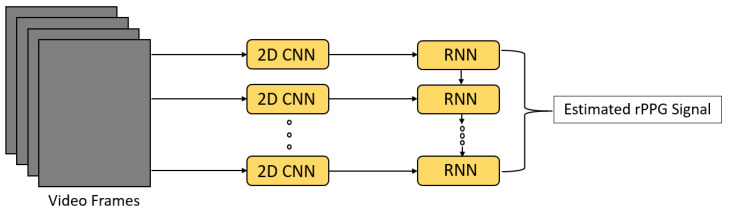
Architecture of RNN-based PhysNet [29].

In the same work of Reference [29], a different version of PhysNet, which combined a 2D CNN with different RNNs (LSTM, BiLSTM, ConvLSTM [40]) was proposed to compare the performance of 3D CNN-based PhysNet and RNN-based PhysNet and evaluate the performance of different RNNs (Figure 9). The input and output of the network remained the same as for the 3D CNN PhysNet. The input was firstly fed into a 2D CNN to extract spatial features of the RGB video frames; then, the RNN was used to propagate these spatial features in the temporal domain. In their research, 3D CNN-based PhysNet achieved a better performance than RNN-based PhysNet, and the BiLSTM variant had the worst performance, indicating the backward information flow of spatial features was not necessary. Table 2 shows the performance of different versions of PhysNet in terms of root mean square error (RMSE) and Pearson correlation coefficient (R) [29].

In Reference [35], another combination of 2D CNN with a ConvLSTM network with attention mechanism was proposed for rPPG signal extraction. The 2D CNN part had a similar approach as DeepPhys [27], which consisted of a trunk branch and a mask branch. The trunk branch was used to extract spatial features from a sequence of face images, while the mask branch learned and generated attention masks and passed them to the trunk branch to guide feature extraction. These spatial features were then fed into a ConvLSTM network in order to make use of the temporal correlation held in video frames for rPPG signal extraction.

## 3. Hybrid Deep Learning Methods

In this section, we describe hybrid DL methods for remote HR measurement. For hybrid DL methods, DL techniques are only applied in some parts of the pipeline. We further indicate whether the methods are used for signal optimization, signal extraction, or HR estimation (Figure 3).

### 3.1. Deep Learning for Signal Optimization

In most existing remote HR measurement pipelines, the input is the original video recorded by a digital camera. Therefore, face detection or skin segmentation is needed to ignore irrelevant background information. Moreover, some specific skin regions, such as the cheeks, contain stronger signals and are usually selected as the ROI [42]. In this subsection, we describe these DL-based signal optimization methods to enable more effective signal extraction.

In Reference [43], a 2D CNN for skin detection was created and trained on a private video database. Both skin and non-skin region samples were manually segmented and treated as positive and negative samples, respectively. Conventional rPPG algorithms (ICA and PCA) were then performed on the detected skin region for evaluation. Tang et al. [43] suggested that low-cost cameras could capture rPPG signals with their method, which worked on single-channel input by choosing the RGB channel with the least noise under different conditions. This method could be combined with traditional rPPG methods in order to improve their performance. However, it utilized all the skin areas of the face for rPPG signal extraction, which may include unnecessary noise. Moreover, their method was only validated on a private dataset with yellow skin tones.

In Reference [44], a single-photon avalanche diode (SPAD) camera was used to record videos. This camera was able to work well in dark environments. The recorded frame was a low-resolution grayscale image. A 2D CNN encoder-decoder model took this as input and produced a single channel image with values between zero and one, representing the probability that the particular pixel was regarded as skin. In addition, a transfer learning approach was adopted in the training process due to the lack of data for this specific skin detection problem. The model was trained on a large dataset of unlabeled face images for colorization and then further trained on a skin mask dataset. Finally, a binary skin mask was obtained by thresholding and passed for signal extraction.

**Figure 10:**
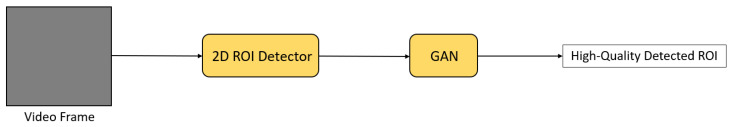
Architecture used in Deep-HR [45] for signal optimization.

In Deep-HR [45], a receptive field block (RFB) network was utilized to detect the ROI as an object [46]. This network was trained on a private dataset with videos recorded in realistic settings to improve overall robustness. Furthermore, a generative adversarial network (GAN)-style module was designed to enhance the detected ROI. A CNN that learned the distribution of high-quality ROIs acted as a discriminator to supervise another deep encoder-decoder network that served as a generator to regenerate the detected ROI. This high-quality detected ROI was passed for subsequent signal extraction. The architecture used for signal optimization in Deep-HR [45] is illustrated in Figure 10.

### 3.2. Deep Learning for Signal Extraction

Signal extraction is the most important part in the remote HR measurement pipeline, and it is the leading focus in this research field. Its principal goal is to extract the rPPG signal from videos for HR estimation. In addition, refining the extracted rPPG signal for better HR estimation is a method to improve the estimation accuracy. Researchers have proposed many different DL methods for obtaining a high-quality rPPG signal, and we are going to categorize and describe them based on the type of neural network being used. Table 3 shows neural networks used in different DL-based signal extraction methods.

#### 3.2.1. Long Short-Term Memory (LSTM)

**Figure 11:**
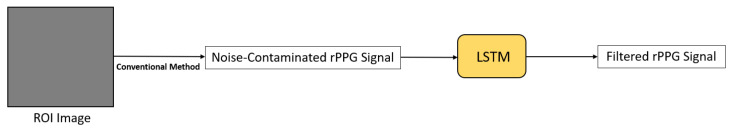
Architecture used in Bian et al. [47].

In References [47,48], a LSTM network was applied for signal filtering, improving the quality of the extracted rPPG signal. As the rPPG signal extracted by conventional methods may contain several noise, filtering the noise-contaminated rPPG signal is able to produce a noiseless rPPG signal for more accurate HR estimation. The LSTM network in Reference [47] was firstly trained on a large amount of synthetic data. Then, it was further trained on real data for model fine-tuning, enhancing its generalization ability. This method is able to effectively overcome the problem of data shortage. The architecture used for signal filtering in Reference [47] is shown in Figure 11.

#### 3.2.2. 2D Convolutional Neural Network (2D CNN)

**Figure 12:**
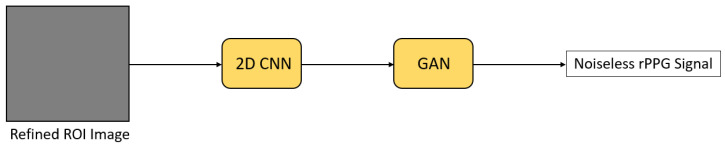
Architecture used in Deep-HR [45] for signal extraction.

In Deep-HR [45], a 2D CNN was learned to extract color information of the ROI pixels (Figure 12). Noise was further removed from the extracted information by using a GAN-style module. A discriminator that accesses high-quality rPPG signals was used to guide a generator to reconstruct a noiseless rPPG signal. This noise removing technique can be applied in other rPPG methods to improve the performance, as well.

MetaPhys [49] utilized a pretrained 2D CNN, namely TS-CAN, which is another version of MTTS-CAN [28] for signal extraction. The difference between them was the use of the multi-task variant so that TS-CAN could only estimate HR and RR one at a time, while MTTS-CAN could estimate HR and RR simultaneously. Furthermore, a meta-learning approach was proposed for better generalization of the model. Model-Agnostic Meta-Learning (MAML) [60] was utilized as the personalized parameter update schema to produce a general initialization so that fast adaptation could be performed when only a few training samples were available. In addition, both supervised and unsupervised training methods were evaluated on MetaPhys. Liu et al. [49] claimed that this approach can reduce bias due to skin tone, improving the robustness of the model.

#### 3.2.3. Spatio-Temporal Network—3D Convolutional Neural Network (3D CNN)

In Reference [50], a 3D CNN was designed to extract features from unprocessed video streams, followed by a multilayer perceptron to regress HR. In the paper, a data augmentation method was also proposed for generating realistic videos effectively with synthetic rPPG signals. The synthetic rPPG signal was transformed to a video by using vector repetition. Noise was also added to the synthetic videos in order to make them realistic.

**Figure 13:**
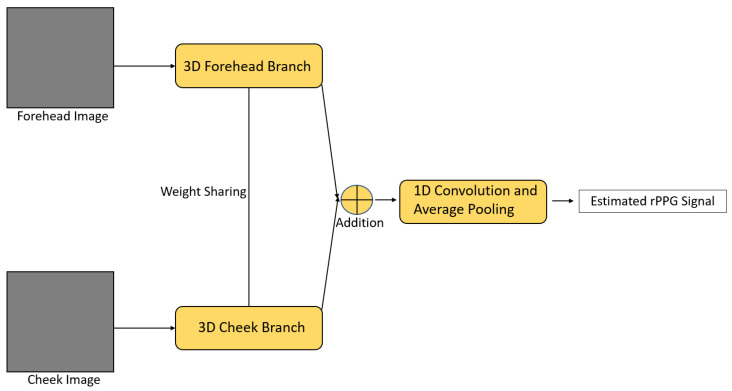
Architecture of Siamese-rPPG [51].

Siamese-rPPG [51] is a framework based on a Siamese 3D CNN (Figure 13). The idea behind this framework is different facial regions may suffer from different noise and have their own appearances. However, they should reflect more or less the same rPPG characteristics. Therefore, the forehead and cheek regions with more rPPG information were firstly selected as the ROI. Next, pixels in these two ROIs were passed to the forehead branch and the cheek branch for extraction, respectively; both were 3D CNNs with the same architecture. Weight sharing mechanism was also applied to these two branches so that, even if either the cheek or forehead region was contaminated with noise, the framework could use the other region for signal extraction, improving the overall robustness. After that, the outputs from these two branches were fused by an addition operation, followed by two 1D convolutional operations and an average pooling, to produce the predicted rPPG signal.

HeartTrack [52] utilized a 3D CNN with attention mechanism for signal extraction. In this 3D spatio-temporal attention network, a hard attention mechanism was used to help the network ignore unrelated background information and a soft attention mechanism was used to help the model filter out covered areas. The extracted signal was further fed into a 1D CNN for time series analysis. Synthetic data was also used in the training process in order to address the problem of inadequate real data.

In Reference [53], a multi-task framework was proposed for learning a rPPG signal extraction model and augmenting data simultaneously. There were a total of 3 main networks in this framework. The first one was a signal extractor that directly extracted the rPPG signal from the input facial videos. The second one was a reconstruction network for generating synthetic videos from real images. The third one was also a reconstruction network for generating synthetic videos from real videos. They were designed to support each other, and these two reconstruction networks could effectively handle the problem of insufficient training data and improve the overall robustness.

DeeprPPG [54] is a framework that can use different skin regions as the input for rPPG signal measurement, allowing customized ROI selection and wider applications. It took a skin region clip from the original video as the input and a spatio-temporal network was utilized to extract the rPPG signal. A spatio-temporal aggregation function was also proposed for easing the side effect of regions contaminated by different noise and improving the robustness of the model.

#### 3.2.4. Spatio-Temporal Network—2D Convolutional Neural Network + Recurrent Neural Network (2D CNN + RNN)

In Reference [55], a two-stream approach was adopted for feature extraction and rPPG signal extraction. For the feature extraction stream, a 2D CNN with low-rank constraint loss function was proposed to force the network to learn synchronized spatial features from spatio-temporal maps, improving the robustness of face detection and ROI alignment errors. For the rPPG signal extraction stream, a 2D CNN was firstly used to extract the rPPG signal, and then the rPPG signal was further refined by a two-layer LSTM network. Lastly, the outputs from these two streams were concatenated for HR estimation.

In Reference [56], a 2D CNN was used to extract spatial features and local temporal information, and an LSTM network was utilized for extracting global temporal information held in consecutive frames. One fully connected layer was further applied to the output of the LSTM to estimate HR. This framework was able to overcome processing latency and update HR in about 1 s, showing the potential of being adopted in real-time HR monitoring.

**Figure 14:**
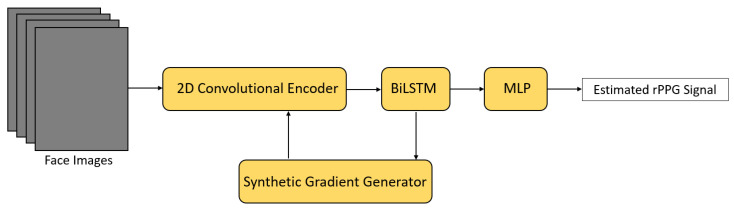
Architecture of Meta-rPPG [57].

Meta-rPPG [57] utilized a transductive meta-learner to take unlabeled data during deployment for self-supervised weight adjustment, allowing fast adaptation to different distribution of samples (Figure 14). In this framework, a ResNet-alike convolutional encoder was firstly used to extract latent features from a stream of face images. Next, these extracted features were passed to a BiLSTM network to model the temporal context, followed by a multilayer perceptron (MLP) for rPPG signal estimation. A synthetic gradient generator was also proposed for transductive learning. It was based on a shallow Hourglass network [61] and further applied to a few-shot learning framework in order to generate gradients for unlabeled data [62].

#### 3.2.5. 3D Convolutional Neural Network + Recurrent Neural Network (3D CNN + RNN)

**Figure 15:**
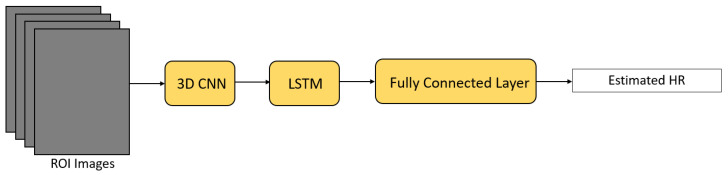
Architecture of PRNet [58].

PRNet [58] is a one-stage spatio-temporal framework for HR estimation from stationary videos (Figure 15). Firstly, a 3D CNN extractor was utilized to extract spatial features and capture local temporal features from the defined ROI. Next, the output feature map was further fed into an LSTM extractor for extracting global temporal features. Lastly, a fully connected layer was applied to estimate HR from the extracted feature map. Huang et al. [58] claimed that this framework is able to predict HR with only 60 frames of the video (2 s), while other remote HR estimation methods usually need 6–30 s of the video.

#### 3.2.6. Generative Adversarial Network (GAN)

**Figure 16:**
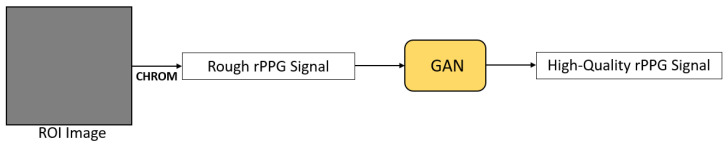
Architecture of PulseGAN [59].

PulseGAN [59] is a framework based on GAN to generate realistic rPPG signals (Figure 16). In the paper, a rough rPPG signal was firstly obtained by applying the CHROM algorithm on the defined ROI. Then, PulseGAN took this as input and generated a high-quality, realistic rPPG signal for performing HR estimation accurately. Moreover, the structure of PulseGAN was based on the conditional GAN approach [63]. The discriminator accessed the ground truth rPPG signal and guided the generator to map a rough rPPG signal extracted by CHROM to a final rPPG signal that is similar to the ground truth one. The rough rPPG signal was also set as a condition in the discriminator. Song et al. [59] mentioned that this framework can be combined with other conventional rPPG methods easily in order to improve the quality of the extracted rPPG signal, resulting in more accurate HR estimation.

### 3.3. Deep Learning for Heart Rate Estimation

Traditionally, the extracted rPPG signal can be filtered with a bandpass filter followed by frequency analysis or peak detection to estimate HR. However, HR estimation can also be classified as a regression problem and solved by DL methods. Moreover, different representations of the HR signal have been proposed for DL-based HR estimation (Table 4).

**Figure 17:**
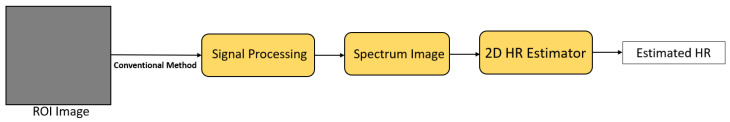
Architecture used in Yang et al. [64].

In Reference [64], the rPPG signal was extracted by conventional methods (e.g., ICA, PCA, CHROM), and short-time Fourier transform and bandpass filtering were applied to the extracted rPPG signal to obtain a frequency domain representation. This representation was further combined with the time domain signal to form a spectrum image, a kind of HR signal representation. Lastly, an HR estimator based on ResNet18 [65] pretrained with the ImageNet dataset was used to estimate HR from spectrum images directly. Based on this method, HR can be estimated accurately regardless of which conventional methods were used, since the HR estimator can learn features in spectrum images and directly map them into HR. The architecture used for HR estimation in Reference [64] is illustrated in Figure 17.

**Figure 18:**
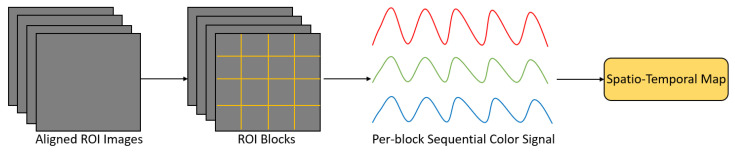
General procedure of constructing a spatio-temporal map. Firstly, the images are aligned and ROI selection is performed to obtain ROI images. Then, these ROI images are divided into several ROI blocks. Next, within each block, the average color value is calculated for each color channel. After that, the average color value of each channel at the same block but different frames are concatenated into temporal sequences. Finally, the temporal sequences of each block are placed into rows to form a spatio-temporal map.

**Figure 19:**
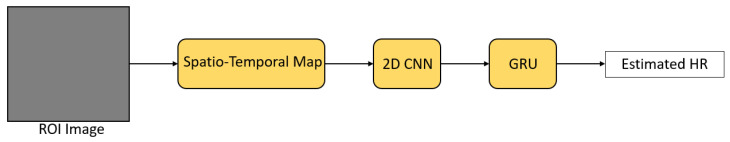
Architecture of RhythmNet [68].

Another type of HR signal representation is the spatio-temporal map (Figure 18) used for HR estimation in References [66,67,68,69,70,71]. Generally, an ROI selection step was involved in the construction of these spatio-temporal maps. Color information of the RGB channels of the ROI pixels was utilized and concatenated in temporal sequences, and placed into rows to form a spatio-temporal map. Finally, a neural network was used to estimate HR from spatio-temporal maps directly. This kind of HR signal representation can highlight the HR signal and suppress the information that is unrelated to the HR signal. In References [66,70], transfer learning was applied to pretrain the HR estimator with the ImageNet dataset to deal with insufficient data. In Reference [68], a combination of 2D CNN and gated recurrent unit (GRU) was used for HR estimation (Figure 19). In Reference [71], NAS was also utilized to find a lightweight and optimum CNN to estimate HR from spatio-temporal maps. In Reference [67], an attention module was added to mitigate the effect of different noise.

**Figure 20:**
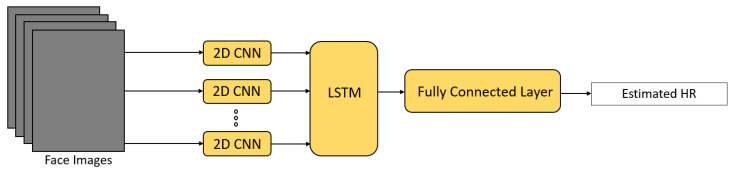
Architecture used in Huang et al. [56].

HR estimation can be treated as a regression problem by using simple fully-connected layers or feedforward neural networks. In References [45,55,56,58], HR was regressed by fully-connected layers from the extracted rPPG signal. The architecture used for HR estimation in Reference [56] is shown in Figure 20. In References [50,52], feedforward neural networks were also utilized to estimate HR from the extracted features.

## 4. Applications

With further research and inevitable technological advances, remote health monitoring technology will undoubtedly play a vital role in many aspects. The utilization of contactless HR monitoring introduces benefits that existing contact-based PPG methods lack. In this section, we describe a few potential applications enabled by remote monitoring of physiological signals.

### 4.1. Affective Computing

Since rPPG technology can be integrated with consumer-level cameras, it has great potential for affective computing and human–computer interaction applications. Researchers have demonstrated the feasibility of rPPG-based methods for interpreting human affects, such as cognitive stress estimation [72,73], emotion recognition [29,74], engagement detection [75], and pain recognition [76,77,78]. These studies illustrate the capability of using rPPG technology beyond the medical domain.

### 4.2. Pandemic Control

With the current COVID-19 outbreak, the value of contactless HR monitoring has become very clear, particularly for screening the public. It has been reported that temperature screening alone is an insufficient indication for coronavirus infection [79,80]. Therefore, the accuracy of screening based on temperature decreases because asymptomatic infected patients have a temperature within the normal range [81,82]. Given this inadequacy, using HR as a criterion for COVID-19 screening was investigated. In References [83,84], it was shown that tachycardia(high HR) is also a symptom of COVID-19. Moreover, the relationship between atrial fibrillation (AF) and COVID-19 was observed in several studies [85,86,87], suggesting rPPG-based AF detection would be useful for discovering potential COVID-19 patients [88]. Meanwhile, during and since the pandemic, the use of wearable smart devices for measuring vital signs, such as HR, BP, and SpO2, have become widespread [89,90]. Such contact-based methods can be replaced by rPPG technology to provide convenience to users with precise screening and detection, resulting in more efficient and effective pandemic control.

### 4.3. Deepfake Detection

Recently, deepfake, a technology to produce high-synthetic videos with DL implementations, has attracted researchers’ attention. Unfortunately, if not tragically, this technology has been used to generate fake news and hoax videos, posing threats to the society. For example, a high-quality video of the 44th President of the United States Barack Obama has been synthesized by using a DL approach [91], which shows him apparently making a speech that he never actually made. These fake videos are of such high quality such that they are indistinguishable to humans and even complicated computer vision algorithms [92,93,94]. As a result, deepfake detection methods need to be developed to encounter such problems. Currently, there have been few attempts in capturing abnormalities in biological signals, such as HR, as a means to detect deepfake videos [95,96].

### 4.4. Telehealth

Within the last few years, telehealth has become more popular all over the world, with more than half of the health care organizations in the U.S. making use of the service [97]. The integration of telehealth with rPPG technology provides various benefits to the users and society. For instance, users will experience a better daily workflow since the time required to travel to healthcare institutions for health-checkups and for doctor consultations will be reduced. Furthermore, the application of telehealth software intertwined with rPPG technology allows the user to measure their physiological signs and detect early symptoms of different health problems (e.g., atrial fibrillation) from any location by using a consumer-level device [88,98,99]. Deploying rPPG technology promotes social distancing and safety for those healthcare workers at the front lines. Furthermore, remote health monitoring can reduce the workload of hospitals and minimize the chance of spreading diseases since it encourages less physical contact with patients and fewer human resources are needed [100], which is especially vital during the midst of a pandemic.

### 4.5. Face Anti-Spoofing

Today, using biometric information of individuals for authentication is very common. One of the most common forms is facial recognition, which is based on the analysis of unique features of a person’s face [101]. However, biometric presentation attacks can exist alongside the face authentication process. For example, attackers can source photos (photo attacks) or videos (replay attacks) of the person from social networking sites easily and present them to the authentication system [102]. Remote HR measurement technology can be incorporated to enhance the authentication system [103]. In References [104,105,106,107,108], rPPG-based face presentation attack detection approaches were developed, suggesting the potential of rPPG technology in the security industry.

### 4.6. Driving Condition Monitoring

In order to reduce the number of traffic accidents, rPPG technology can be adopted to monitor drivers and track a driver’s physiological status. Most road accidents are caused by human factors including fatigue, drowsiness, and illness. Factors, such as disparity in oxygen levels, HR, and RR, may lead to non-specific health problems, which interfere with or degrade decision-making capabilities. This monitoring allows abnormal vital signs to be detected early, with alerts shown immediately so that drivers can adjust their behavior accordingly, avoiding accidents. There have been several attempts for monitoring drivers’ physiological conditions using rPPG methods [109,110,111,112,113,114,115,116,117,118]. In References [112,116,118], a near-infrared camera was used instead of an RGB camera for monitoring. In References [113,115], neural networks were applied for physiological signal estimation.

### 4.7. Searching for Survivors during Natural Disasters

During natural disasters, such as earthquakes and fires, searching for survivors becomes a vital but extremely challenging task. Rescue teams must operate in extremely hazardous conditions, such as collapsed buildings. rPPG technology can be a potential way to reduce risk for search and rescue teams, and improve their efficiency. In References [119,120,121], an unmanned aerial vehicle (UAV) or a drone was used to capture videos, representing a more convenient, safe, and effective way to look for survivors. In Reference [122], research using a drone for multiple subject detection over a long-distance was conducted. This illustrates the potential of using controllable devices equipped with a camera combined with rPPG technology for searching for survivors.

### 4.8. Neonatal Monitoring

As neonates or infants have very sensitive and fragile skin, using contact-based methods to measure their health conditions is inappropriate. rPPG methods are one of the suitable candidates for long-term physiological status monitoring of newborns in neonatal intensive care units (NICU). Several studies have trialed rPPG methods for such monitoring [9,123,124,125,126,127,128,129,130,131,132]. In Reference [131], DL-based segmentation was utilized to reduce computational time, which brought it one step closer to real-time applications. In Reference [132], DL-based ROI detection was applied to handle pose variations and illumination changes, further improving the estimation accuracy. These examples indicate the promise of using rPPG technology for neonatal monitoring.

### 4.9. Fitness Tracking

During fitness training, having health monitors to keep track of the current physiological condition is an excellent way to prevent over-exercising and help to adjust the fitness process to an individual’s real-time needs and condition. Contact-based methods, such as smartwatches or digital bracelets, for such monitoring can cause discomfort or pain during heavy exercise. rPPG technology can be utilized to provide simple remote fitness tracking. In References [20,133,134,135,136], rPPG methods in fitness training settings were studied. In References [20,133,136], motion artifact during exercise was the major focus. In References [134,135], a feedback control system was implemented, as well, for adjusting the speed of the treadmill automatically.

## 5. Resources

As remote physiological monitoring is an emerging field in computer vision and biomedical engineering, there are resources available for researchers to accelerate progress and ease the transition of newcomers. In this section, we detail some of the open-source toolboxes to help to implement related algorithms and most of the datasets that are commonly used for model training and benchmarking. Furthermore, open challenges in rPPG are also described to encourage various researchers to contribute to the field.

### 5.1. Toolboxes

iPhys [137] is an open-source toolbox written in MATLAB. It contains commonly used implementations in rPPG pipelines, such as face detection, ROI definition, and skin segmentation. It also includes four conventional rPPG methods for baseline comparison. Other plotting and signal quality calculation functions are provided, as well, for performance evaluation.

In Reference [138], the whole rPPG pipeline based on ICA and some utilities are written in MATLAB. Beginners can quickly run or even modify the provided script to evaluate the performance of the particular rPPG method.

The Python tool for Virtual Heart Rate (pyVHR) [139] is a recently developed Python package for heart rate estimation based on rPPG methods. In this package, 8 conventional rPPG methods are implemented and evaluated based on 5 datasets. Other frequently used pre-processing and post-processing techniques are provided, as well. Practitioners can extend the framework to evaluate their own algorithms on these 5 datasets.

### 5.2. Datasets

Performance evaluation is important for researchers to test whether their proposed methods are good enough when compared with other methods and able to solve existing challenges. For supervised methods, datasets are also crucial for proper training and achieving state-of-the-art performance. In this subsection, we detail most of the datasets that are commonly used for benchmarking and model training.

AFRL [140] is a dataset proposed by the United States Air Force Research Laboratory. It was aimed to evaluate the effect of head motion artifacts. During data acquisition, a multi-imager semicircular array (a total of 9 synchronized, visible spectrum imagers) centered on the imaged participant in a controlled light environment was used to record the participant’s head motions during specific tasks. At the same time, electrocardiogram (ECG) and fingertip reflectance PPG were recorded as ground truth signals. The imaged participant was told to perform specific tasks, which included staying still, sweeping around the imagers with a pre-defined angle per second, and randomly re-orienting the head position to an imager. The background of the environment consisted of either a solid black fabric or a patterned, colored fabric.

COHFACE [141] is a publicly available dataset proposed by the Idiap Research Institute. The purpose of proposing this dataset was to allow researchers to evaluate their developed rPPG algorithms on a publicly available dataset so that comparisons between different algorithms could be conducted in a standard and principled manner. In this dataset, a conventional webcam was used to capture the full face of the participant in two different illumination settings (studio lighting and natural lighting) to evaluate the effect of illumination variation. Skin reflectance PPG and respiratory signal were recorded as ground truth signals. The only disadvantage of this dataset was the heavy compression, so noise artifact was unavoidably added.

MAHNOB-HCI [142] is a multimodal dataset that was originally recorded for emotion recognition and implicit tagging research. However, as ground truth signals, such as ECG and respiration amplitude, were recorded, it was also suitable for rPPG algorithm evaluation. Moreover, six cameras were used to capture different views (frontal view, profile view, wide angle, close ups) of the participant, which made this dataset useful for evaluating the algorithm when pose angle varied.

MMSE-HR [143] is another multimodal dataset proposed for facial expression analysis. Some vital signs, such as BP, RR, and HR, were also recorded, making this dataset appropriate for testing rPPG algorithms. Furthermore, subjects from different races (Black, White, Asian, Hispanic/Latino) participated in the data acquisition, so researchers were able to evaluate their proposed methods against different skin tones.

OBF [41] is a large dataset made by the University of Oulu in Finland specifically for remote physiological signal measurement. Aside from healthy subjects in this dataset, patients with atrial fibrillation (AF) also participated in data collection in order to validate rPPG methods for clinical applications, such as diagnosing cardiac diseases. In addition, there were two different recording states, one for healthy participants and one for AF patients. Healthy participants were recorded in a resting state and a post-exercise state (5-min exercise). AF patients were recorded before and after cardioversion treatment.

PURE [144] is a dataset proposed for examining head motion artifacts in rPPG methods in more detail. During data acquisition, participants were told to perform six different tasks (holding steady, talking, slow translation, fast translation, small rotation, medium rotation) in order to introduce different kinds of head motion. Naturally changing illumination (daylight with clouds through a large window) was used for recording, as well.

UBFC-RPPG [145] is another dataset proposed mainly for rPPG algorithm evaluation. The data recording was conducted indoors with indoor illumination and slight changes in sunlight. One special aspect of the recording is that participants were told to play a time-sensitive mathematical game. Its purpose was to augment the HR of participants and hence simulate a real-life human-computer interaction scenario for evaluation.

VIPL-HR [146] is a large-scale multimodal dataset created for remote pulse estimation research. In this dataset, various face variations due to head motion (stable, large motion, talking), illumination changes (lab, dark, bright), and acquisition diversity (smartphone, webcam, RGB-D camera) were introduced in order to test the overall robustness of the proposed algorithm. The dataset was compressed with different codecs (MJPG, FMP4, DIVX, PIM1, X264) in order to retain the completeness of the signals as much as possible, while being convenient for public access at the same time.

A summary of the mentioned datasets is provided in Table 5. Moreover, Table 6 illustrates the performance of all mentioned DL methods on these common datasets. The evaluation metrics in Table 6 include root mean square error (RMSE) in bpm, mean absolute error (MAE) in bpm, Pearson correlation coefficient (R), and signal-to-noise ratio (SNR) in decibels (dB).

### 5.3. Open Challenge on Remote Physiological Signal Sensing

Creating an open challenge on a specific machine learning task is a common way in the field of machine learning to encourage people to participate and solve a particular problem using DL methods. One of the most famous open challenges is the ImageNet Large Scale Visual Recognition Challenge (ILSVRC) [147]. This challenge has been running annually for 8 years (2010–2017), and its focuses are object recognition, object detection, and image classification. Many DL methods have been proposed for this task, and this competition has definitely boosted research interest in this field, allowing rapid development in DL-based computer vision. An open challenge on remote physiological signal sensing was also organized in 2020, namely Remote Physiological Signal Sensing (RePSS 2020) [148]. In this challenge, the focus was measuring the average HR from color facial videos. The VIPL-HR-V2 dataset, which is the second version of VIPL-HR [146], and the OBF dataset [41] were used for model training and testing. The RePSS 2021 is also currently running, and its focus was changed to measure inter-beat-interval (IBI) curve and RR. This open challenge can have the same optimistic effect as ILSVRC, encouraging people to participate and engage in this research field.

## 6. Research Gaps

During the last few decades, many methods for ascertaining remote HR measurements have been proposed. This has attracted much attention, and increasing numbers of researchers are engaged in this exciting area. In this section, we discuss some of the research gaps in order to suggest some possible future directions in this field.

### 6.1. Influencing Factors

The performance of remote HR measurement based on rPPG is influenced by many factors, such as illumination changes, motion artifacts, skin-tone variations, and video compression [12,14,22,23,149]. There are several methods proposed for handling these challenges. For example, the utilization of different HR signal representations, such as spectrum images [64] and spatio-temporal maps [66,67,68,69,70,71], as well as the use of attention mechanism [27,28,30,32,34,35,49,52,67], can deal with illumination variations and motion noise. STVEN [30] was designed to improve the robustness of HR measurement under video compression. Meta-learning approaches with fast adaptation to uncommon samples [49,57] are suitable to deal with skin-tone variations. Additional work is needed to better understand and quantify the effects these influencing factors have on remote physiological measurement. More importantly, new methods should provide insight into how these challenges are handled from a technical and biophysical perspective, rather than just evaluating their performance on a dataset that contains the influencing factors.

### 6.2. Measuring Other Vital Signs

Undoubtedly, HR is a very important physiological indicator to indicate the current health condition of a person. Researchers in this field are mainly interested in estimating HR, followed by RR. However, other vital signs are also important [150,151]. For example, BP is useful in detecting some cardiovascular diseases, such as hypertension, while SpO2 can reflect the health of the cardiorespiratory system by showing if a person has an adequate supply of oxygen. At the same time, these vital signs are associated to COVID-19, and they are useful for COVID-19 diagnosing, as well [152,153].There are relatively fewer studies that attempt to estimate BP [154,155,156] and SpO2 [157,158,159] remotely when compared HR and RR. There are still many research opportunities in other vital signs.

### 6.3. Datasets

Datasets are increasingly important for evaluating new proposed methods whether demonstrating success in addressing specific problems or increasing the effectiveness of previously proposed methods. For DL methods, datasets are even more important as they are used for training in supervised methods, as well. The performance of the supervised methods are greatly affected by the training datasets. Currently, most of the existing publicly available datasets focus on two major challenges in rPPG methods only, that is, motion artifacts and illumination variations [12,23]. Other challenges, such as skin-tone variations [22,160,161], multiple persons detection [14,122], and long distance estimations [14,162], need to be overcome, as well, if the methods are to be, ultimately, robust and highly applicable in the real-world, replacing all contact-based methods. Moreover, the subjects in these datasets are mainly adult participants. Datasets with newborns as participants are also desirable for evaluating rPPG methods. As a result, more comprehensive, high diversity and high quality datasets are needed to fully evaluate the robustness of any new proposed method and allow comprehensive training in supervised methods. Such datasets are extremely beneficial to the research community.

### 6.4. Performance on Different Heart Rate Ranges

According to performance results of RePSS 2020 [148], the top 3 teams were able to achieve a significantly better performance on the middle HR level, where HR ranges from 77 to 90 bpm, followed by the low HR level (less than 70 bpm). Performance at the high HR level (more than 90 bpm) was the worst. This is a challenge that absolutely needs to be addressed in order to be accurate enough to be applied in real-world applications because these significantly lower or higher HRs are showing specific health problems. Moreover, this result indicates that using common metrics, such as mean absolute error (MAE), root mean square error (RMSE), signal-to-noise ratio (SNR), and Pearson correlation coefficient (R), to evaluate rPPG methods may not be effective enough. Evaluation on a wider range of HR levels is required in order to comprehensively test the robustness of the proposed method.

### 6.5. Understanding of Deep Learning-Based Methods

The advantage of using CNN in rPPG technology is that a good result can be obtained without very deep understanding or analysis of the specific problem; the disadvantage is that this DL method is a black box, which means we do not have a full understanding of why such a result is obtained. The lack of understanding of how CNN-based methods work on rPPG technology may be a barrier to further development of this technology and evaluation of these DL methods. Reference [25] is a work that focused on the understanding of CNN-based rPPG methods, rather than proposing a new model with state-of-the-art performance. Several experiments were performed to explore the CNN-based rPPG signal extraction and improve the understanding of this approach. In the paper, some important observations have been made. For example, it showed that the CNN for rPPG signal extraction is actually learning information related to the PPG signal but not the motion-induced intensity changes [27]. In addition, the CNN training is affected by the physiological delay between the video data and the reference finger oximeter. Researchers should direct their attention to more studies that focus on the understanding of DL-based rPPG methods in order to gain valuable insights and further improve the performance of these DL approaches.

## 7. Conclusions

In recent years, many methods for remote HR measurement have been proposed. Due to rapid development in the area of machine learning, DL methods have shown significant promise in this field. In this paper, we have provided a comprehensive review on most of the existing recent DL-based methods for remote HR estimation. We have further categorized these methods into end-to-end and hybrid DL methods, and grouped them based on the type of neural network being used. We then described some potential applications that can be achieved by using rPPG technology. Next, some rPPG resources, such as toolboxes, datasets, and open challenges, have been detailed in order to help accelerate research. Lastly, we have discussed some of the current research gaps in this field to shed some light on future areas and directions in this exciting field.

As remote physiological measurement establishes itself as an emerging research field, we suggest more work should focus on addressing different influencing factors and estimating other vital signs, which will assist in bridging the gap for real-world applications. Furthermore, high-quality and diverse datasets are crucial for proper benchmarking and analysis of different methods and the future development of more complex DL models and architectures. Last but not least, the understanding of different DL-based approaches is critical, especially when integrating these networks for high-stakes applications, such as healthcare diagnostics.

## Figures and Tables

**Table 1:** Summary of end-to-end deep learning (DL) methods for remote HR measurement.

Ref.	Year	2D CNN	3D CNN	2D CNN + RNN	NAS	Attention
[26]	2018	✓				
[27]	2018	✓				✓
[28]	2020	✓				✓
[29]	2019		✓	✓		
[30]	2019		✓			✓
[31]	2020		✓		✓	
[32]	2021		✓			✓
[33]	2021		✓			
[34]	2021		✓			✓
[35]	2019			✓		✓

**Table 2:** Performance of different versions of PhysNet [29] on the OBF [41] dataset. Root mean square error (RMSE) in beats per minute (bpm) and Pearson correlation coefficient (R) were used as the evaluation metrics.

3D CNN-Based	LSTM Variant	BiLSTM Variant	ConvLSTM Variant
RMSE = 2.048, R = 0.989	RMSE = 3.139, R = 0.975	RMSE = 4.595, R = 0.945	RMSE = 2.937, R = 0.977

**Table 3:** Summary of hybrid DL methods for signal extraction in remote HR measurement pipeline.

Ref.	Year	LSTM	2D CNN	3D CNN	2D CNN + RNN	3D CNN + RNN	GAN
[47]	2019	✓					
[48]	2020	✓					
[45]	2020		✓				✓
[49]	2021		✓				
[50]	2019			✓			
[51]	2020			✓			
[52]	2020			✓			
[53]	2020			✓			
[54]	2020			✓			
[55]	2019				✓		
[56]	2020				✓		
[57]	2020				✓		
[58]	2021					✓	
[59]	2021						✓

**Table 4:** Summary of all mentioned end-to-end and hybrid DL methods for remote HR measurement.

Ref.	Year	End-to-End/Hybrid	Description
[26]	2018	End-to-End	End-to-end HR estimation with an extractor and an estimator
[27]	2018	End-to-End	Normalized frame difference as motion representation,
			attention mechanism was used to guide the motion model,
			visualization of spatio-temporal distribution of physiological signals
[43]	2018	Hybrid	2D CNN network for skin detection
[64]	2018	Hybrid	Spectrum images were used for HR estimation
[66]	2018	Hybrid	Spatio-temporal maps were used for HR estimation,
			transfer learning approach to deal with data shortage
[29]	2019	End-to-End	Compared 3D CNN-based and RNN-based spatio-temporal network,
			can estimate HR and HRV accurately
[30]	2019	End-to-End	Enhancing video quality to deal with highly compressed videos,
			can estimate HR and HRV accurately
[35]	2019	End-to-End	Attention mechanism was used to guide the trunk branch for signal extraction
[47]	2019	Hybrid	LSTM network for signal filtering,
			transfer learning approach to deal with data shortage
[50]	2019	Hybrid	3D CNN for signal extraction,
			data augmentation method for generating videos with synthetic rPPG signals,
			multilayer perceptron for HR estimation
[55]	2019	Hybrid	2D CNN-based two-stream approach for signal extraction,
			and LSTM network for signal refining
[67]	2019	Hybrid	Spatio-temporal maps were used for HR estimation,
			attention mechanism was applied to remove noise
[28]	2020	End-to-End	Temporal shift module to model temporal information,
			attention mechanism was applied to guide the motion model,
			able to estimate HR and RR simultaneously by one network
[31]	2020	End-to-End	Used NAS to find a well-suited network for HR estimation
[44]	2020	Hybrid	2D CNN encoder-decoder model for skin detection,
			transfer learning approach to deal with data shortage
[45]	2020	Hybrid	Two GAN-style modules to enhance the detected ROI and remove noise,
			2D CNN for signal extraction
[48]	2020	Hybrid	LSTM network for signal filtering
[51]	2020	Hybrid	Siamese 3D CNN for signal extraction
[52]	2020	Hybrid	3D CNN with attention mechanism for signal extraction,
			feedforward neural network for HR estimation
[54]	2020	Hybrid	3D CNN that can take different skin regions for signal extraction
[56]	2020	Hybrid	2D CNN + LSTM spatio-temporal network for signal extraction
[57]	2020	Hybrid	2D CNN + BiLSTM spatio-temporal network for signal extraction,
			meta-learning approach for fast adaptation
[68]	2020	Hybrid	Spatio-temporal maps were used for HR estimation
[69]	2020	Hybrid	Spatio-temporal maps were used for HR estimation
[70]	2020	Hybrid	Spatio-temporal maps were used for HR estimation,
			transfer learning approach to deal with data shortage
[32]	2021	End-to-End	Avoid extracting redundant information from video segments,
			attention mechanism was applied to deal with different noise
[33]	2021	End-to-End	An efficient framework for performing HR estimation quickly
[34]	2021	End-to-End	Dealt with the problem of extracting redundant video information,
			attention mechanism was applied to learn important features and eliminate noise
[49]	2021	Hybrid	TS-CAN from another paper was utilized for signal extraction,
			meta-learning approach for fast adaptation
[53]	2021	Hybrid	Multi-task framework for simultaneous signal extraction and data augmentation
[58]	2021	Hybrid	3D CNN + LSTM spatio-temporal network for signal extraction
[59]	2021	Hybrid	GAN for generating high-quality rPPG signal from rough rPPG signal
[71]	2021	Hybrid	Spatio-temporal maps were used for HR estimation,
			NAS was used to find a CNN for mapping spatio-temporal maps into HR

**Table 5:** Summary of common datasets for remote physiological monitoring.

Dataset	Subjects	Description
AFRL [140]	25	9 RGB cameras with 120 fps, resolution is 658 × 492, ECG, PPG, RR are recorded
COHFACE [141]	40	1 RGB webcam with 20 fps, resolution is 640 × 480, BVP, RR are recorded
MAHNOB-HCI [142]	27	1 RGB camera with 60 fps, 5 monochrome cameras with 60 fps, both resolution are 780 × 580, ECG, RR are recorded
MMSE-HR [143]	140	1 3D stereo imaging sensor with 25 fps, 1 2D video sensor with 25 fps, 1 thermal sensor with 25 fps, RGB sensor resolution is 1040 × 1392, thermal sensor resolution is 640 × 480, HR, RR, BP are recorded
OBF [41]	106 (6 with atrial fibrillation)	1 RGB camera with 60 fps, 1 NIR camera with 30 fps, RGB camera resolution is 1920 × 1080, NIR camera resolution is 640 × 480, ECG, BVP, RR are recorded
PURE [144]	10	1 RGB camera with 30 fps, resolution is 640 × 480, HR, SpO2, PPG are recorded
UBFC-RPPG [145]	42	1 RGB webcam with 30 fps, resolution is 640 × 480, HR, PPG are recorded
VIPL-HR [146]	107	1 RGB webcam with 25 fps, 1 RGB-D camera with 30 fps, 1 smartphone camera with 30 fps, RGB webcam resolution is 960 × 720, RGB-D NIR camera resolution is 640 × 480, RGB-D RGB camera resolution is 1920 × 1080, smartphone camera resolution is 1920 × 1080, HR, SpO2, BVP are recorded

**Table 6:** Performance of all mentioned end-to-end and hybrid DL methods for HR measurement on commonly used datasets listed in Table 5. Refs. [43,44,56] are not included here as they are evaluated on their own private datasets.

Methods	AFRL	COHFACE	MAHNOB-HCI	MMSE-HR	OBF	PURE	UBFC-RPPG	VIPL-HR
[26]	X	RMSE = 10.78	RMSE = 9.24	X	X	RMSE = 2.37	X	X
		MAE = 8.10	MAE = 7.25			MAE = 1.84		
		R = 0.29	R = 0.51			R = 0.98		
[27]	MAE = 2.45	X	MAE = 4.57	X	X	X	X	X
	SNR = 4.65		SNR = −8.98					
[64]	X	X	RMSE = 4.26	X	X	X	X	X
			R = 0.81					
[66]	X	X	RMSE = 4.49	RMSE = 6.83	X	X	X	X
[29]	X	X	RMSE = 7.88	X	RMSE = 1.812	X	X	X
			MAE = 5.96		R = 0.992			
			R = 0.76					
[30]	X	X	RMSE = 5.93	X	RMSE = 1.8	X	X	X
			MAE = 4.03		R = 0.992			
			R = 0.88					
[35]	X	RMSE = 11.88	X	X	X	RMSE = 1.58	X	X
		MAE = 7.31				MAE = 0.88	X	X
		R = 0.36				R = 0.99	X	X
		SNR = −1.93				SNR = 9.18	X	X
[47]	X	X	X	RMSE = 3.187	X	X	X	X
				MAE = 4.35				
				R = 0.8254				
[50]	X	X	X	X	X	X	RMSE = 8.64	X
							MAE = 5.45	X
[55]	X	RMSE = 9.96	X	X	X	X	X	X
		MAE = 8.09						
		R = 0.40						
[67]	X	X	X	RMSE = 10.10	X	X	X	RMSE = 7.99
				R = 0.64				MAE = 5.40
								R = 0.66
[28]	RMSE = 3.72	X	X	RMSE = 5.66	X	X	X	X
	**MAE = 1.45**			MAE = 3.00				
	R = 0.94			**R = 0.92**				
	**SNR = 8.64**			SNR = 2.37				
[31]	X	X	RMSE = 5.10	RMSE = 5.87	X	X	X	RMSE = 8.68
			MAE = 3.78	R = 0.89				MAE = 5.68
			R = 0.86					R = 0.72
[45]	X	X	RMSE = 3.41	X	X	X	X	X
			R = 0.92					
[48]	X	X	X	**MAE = 1.31**	X	X	X	X
				**SNR = 9.44**	X	X	X	X
[51]	X	**RMSE = 1.29**	X	X	X	RMSE = 1.56	**RMSE = 0.97**	X
		MAE = 0.70				MAE = 0.51	MAE = 0.48	
		R = 0.73				R = 0.83		
[52]	X	X	X	X	X	X	RMSE = 3.368	X
							MAE = 2.412	
							**R = 0.983**	
[54]	X	RMSE = 7.06	RMSE = 6.26	X	X	**RMSE = 0.43**	X	X
		MAE = 3.07	MAE = 4.81			**MAE = 0.28**		
		**R = 0.86**	R = 0.79			R = 0.999		
[57]	X	X	RMSE = 3.68	X	X	X	RMSE = 7.42	X
			MAE = 3.01				MAE = 5.97	
			R = 0.85				R = 0.53	
[68]	X	X	RMSE = 3.99	RMSE = 5.49	X	X	X	RMSE = 8.14
			R = 0.87	R = 0.84				MAE = 5.30
								R = 0.76
[69]	X	X	X	RMSE = 6.04	**RMSE = 1.26**	X	X	**RMSE = 7.97**
				R = 0.84	**R = 0.996**			**MAE = 5.02**
								**R = 0.796**
[70]	X	X	**RMSE = 3.23**	X	X	X	X	X
			**MAE = 1.53**					
			**R = 0.97**					
[32]	X	RMSE = 7.52	X	X	X	RMSE = 1.21	X	X
		MAE = 5.19				MAE = 0.74		
		R = 0.68				**R = 1.00**		
[33]	X	RMSE = 9.50	X	X	X	X	RMSE = 3.82	X
		MAE = 5.57					MAE = 2.15	
		R = 0.75					R = 0.97	
[34]	X	RMSE = 6.65	X	RMSE = 5.84	X	RMSE = 0.77	RMSE = 3.97	X
		MAE = 4.67		R = 0.85		MAE = 0.34	MAE = 1.46	
		R = 0.77				R = 0.99	R = 0.93	
[49]	X	X	X	**RMSE = 3.12**	X	X	RMSE = 3.12	X
				MAE = 1.87			MAE = 2.46	
				R = 0.89			R = 0.96	
[53]	X	RMSE = 1.65	X	X	X	RMSE = 1.07	RMSE = 2.09	X
		**MAE = 0.68**				MAE = 0.40	**MAE = 0.47**	
		R = 0.72				R = 0.92		
[58]	X	X	RMSE = 6.42	X	X	X	RMSE = 7.24	X
			MAE = 5.01				MAE = 5.29	
[59]	X	X	RMSE = 6.53	X	X	RMSE = 4.29	RMSE = 2.10	X
			MAE = 4.15			MAE = 2.28	MAE = 1.19	
			R = 0.71			R = 0.99	R = 0.98	
[71]	X	X	X	X	X	RMSE = 2.02	X	RMSE = 8.01
						MAE = 1.65		MAE = 5.12
						R = 0.99		R = 0.79

## Data Availability

Not applicable.
